# Retractorless Surgery for Petroclival Meningiomas via the Subtemporal Approach: A Try to Reduce Brain Retraction Injury

**DOI:** 10.1155/2022/6436542

**Published:** 2022-07-14

**Authors:** Dongxue Li, Minghui Zeng, Yang Yao, Nan Zhang, Tao Yang, Chengyu Xia

**Affiliations:** ^1^Department of Neurosurgery, The First Affiliated Hospital of USTC, Division of Life Science and Medicine, University of Science and Technology of China, Hefei, Anhui 230036, China; ^2^Bengbu Medical College, Bengbu, Anhui 233000, China; ^3^Intelligent Pathology Institute, The First Affiliated Hospital of USTC, Division of Life Sciences and Medicine, University of Science and Technology of China, Hefei, Anhui 230036, China

## Abstract

**Purpose:**

To present our experience with retractorless surgery for resection of petroclival meningiomas (PCMs) via the subtemporal approach with routine operative instruments.

**Methods:**

Clinical data of patients with PCMs who received surgical treatments via subtemporal approach were retrospectively analyzed. Patient demographics, duration of operation, extent of resection, postoperative brain injury rate, postoperative complication, and surgical outcome were reviewed.

**Results:**

Twenty-nine consecutive patients with retractorless surgery via subtemporal approach performed between November 2018 and November 2021. The gross total resection rate was 82.8% (*N* = 24). The incidence of postoperative temporal lobe injury was 3.4% (*N* = 1). All the procedures were completed without fixed retraction or other specialized instruments.

**Conclusions:**

Retractorless surgery via subtemporal approach is a reliable treatment option for PCMs, which can be completed with routine operative instruments.

## 1. Introduction

Retractorless surgery, an alternative to continuous or intermittent brain retraction, is widely accepted to reduce brain retraction injuries [1–4]. Retractorless surgery was firstly reported by professor Spetzler in 2012 [5], who presented the results of 223 consecutive cases of vascular lesions and skull base tumors and found that more than 90% of the procedures could be completed without fixed retraction. With the careful and accurate use of the handheld suction device and operating instruments, the fixed retraction was replaced by retractorless technique, thus avoiding brain injuries related to a fixed retractor. Since then, the retractorless surgery has been performed in giant vestibular schwannomas, intracranial aneurysm clipping, and deep brain tumor resection [1, 3, 6, 7]. A movement away from the use of fixed retractors has also been heralded as an advance in skull base surgery. However, as showed in the current reports of retractorless surgery [2, 5, 6], a few cases still required the use of a fixed retractor. The retractorless surgery was completed with the assistance of specialized operative instruments such as fiberoptic-lighted instruments and microscope with foot pedals or a mouthpiece, and some skull-base neurosurgeons were still wondering if retractorless surgery can only be performed with chosen patients or if retractorless surgery can only be completed with specialized operative instruments.

In petroclival meningiomas (PCMs), the management remains a formidable technical challenge for the skull base surgeons [8–10]. Complete resection of these benign tumors is the only potentially curative option [10]. However, it is difficult to ensure excellent surgical exposure and complete resection, and sometimes even severe postoperative complications may occur [9–14]. The subtemporal approach, with advantages of exemplary surgical exposure, easy procedure of craniotomy, and less trauma, is widely used for PCM resection [12, 15, 16]. However, temporal lobe injury was significantly associated with the subtemporal approach [14, 17, 18]. To reduce temporal lobe injury related to brain manipulation or retraction remains one of the essential goals in such an approach [19].

So far as we know, there is still no report of retractorless surgery for PCMs. We thus propose the following questions: can retractorless surgery be completed—when performed on PCMs, the king cobra of all skull base tumors [18], with routine operative instruments? If so, how to minimize any form of brain manipulation? In this article, we reported our results and technical details of retractorless surgery for PCM resection with routine operative instruments, focusing on the subtemporal approach and postoperative temporal lobe injury.

## 2. Methods

### 2.1. Patients

This study was conducted in the Department of Neurosurgery, the First Affiliated Hospital of the University of Science and Technology of China (Anhui Provincial Hospital). As a retrospective study, the ethics committee of our hospital approved it to be exempt requiring informed consent from the patients.

Twenty-nine consecutive patients received surgical treatments via subtemporal approach during November 2018 and November 2021, which were enrolled in this study. Clinical data including patient demographics, tumor size, preoperative symptoms, duration of symptoms, size of tumors, procedure time, extent of resection, incidence and degree of postoperative brain injury, postoperative complications, and KPS of three months after the surgery were collected. All patients underwent preoperative magnetic resonance image (MRI) and immediately postoperative computed tomography (CT) scan or MRI scan within 72 hours to check for any new cerebral injury. Retraction injury was diagnosed when the retraction site's high signal intensity was present on postoperative T2WI.

According to the MRI and CT finding in one week postoperatively, we classified the temporal lobe injury as follows. Grade I: the high signal intensity on T2WI, CT was negative. Grade II: hyperdensity on T2-weighted imaging and hypodensity on CT scan were observed on the retraction site. Grade III: temporal lobe contusion or infarction on the retraction site. Grade IV: intracerebral hematoma (more than 5 ml) was discovered adjacent to the retraction site. However, the medial temporal lobe injury was not considered as retraction-related brain injury, as it may be caused by resection of the tumor on the medial temporal lobe.

### 2.2. Strategies of Retractorless Surgery

A lumbar spinal catheter was routinely employed for cerebrospinal fluid (CSF) drainage before positioning except for the patient with preoperative obstructive hydrocephalus. Mannitol was only used when a lumbar drain was contraindicated or significantly increased intracranial pressure was present. Patients were placed in the lateral position with the zygomatic arch horizontal to the floor. The incision began from the tragus, extended perpendicular to the zygomatic arch, turned a little anterior on the pinna's upper edge, and continued approximately to the superior temporal line level. Fascia and temporal muscle were exposed, split, and then retracted with fishhooks. A craniotomy with a diameter of 4 cm, centered in the root of the zygomatic arch, was performed. The bone window's inferior margin should be down to the bottom of the middle cranial fossa to minimize the retraction of the temporal lobe. The mastoid air cell, if it was opened during the craniotomy, should be sealed completely with bone wax.

**Figure 1:**
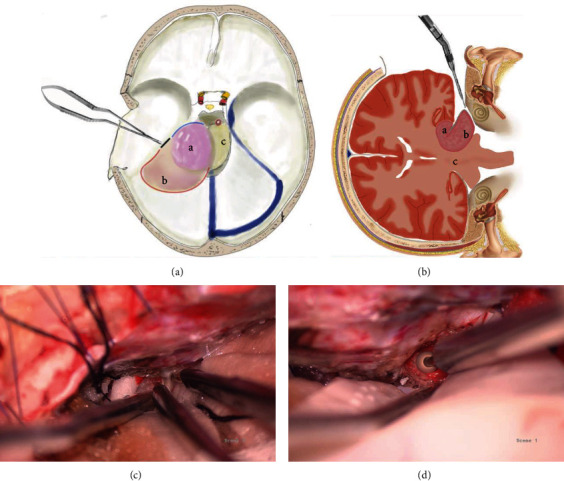
The surgical strategy of retractorless technics. (a) A linear incision was made on the tentorium of the tumor's attachment (the black line); the tentorium incision was then extended along the tumor's posterior margin to the perimesencephalic cistern (the blue line) and along with the petrous erosion to the anterior margin of the tumor (the red line). A: tumor above the tentorium; B: tumor under the tentorium; C: brain stem. (b) The incision on the tentorium may cut off the tumor's blood supply with minimal retraction of the temporal lobe. Then remove the petrous erosion in the same corridor with a high-speed drill for better tumor exploration. (c) The intraoperative image shows an incision was made on the tentorium without a fixed retractor. (d) The intraoperative image shows the petrous erosion is being removed with a high-speed drill for better tumor exploration.

Before opening the dura mater, hyperventilation was performed to decrease the intracranial pressure (ICP) and obtain a larger corridor. During the procedure, the arterial partial pressure of carbon dioxide (PaCO2) was supposed to be maintained at approximately 28 mmHg. Open the dura in a U-shaped fashion, and pay great attention to protect the vein of Labbé. After the appropriate positioning, the release of CSF by lumbar spinal catheter, and hyperventilation, the temporal lobe can be easily elevated with handheld suction to expose the middle cranial fossa base and tentorium. The tumor does not need to be fully but properly exposed. A linear incision was made on the tentorium attachment. The tentorium incision was then extended along the posterior margin of the tumor to the perimesencephalic cistern and along with the petrous erosion to the anterior margin of the tumor (Figure 1(a)) to cut off the tumor's blood supply with minimal retraction of the temporal lobe. A high-speed drilling or piezoelectric scalpel was then used to remove the bone of the petrous apex to gain an unobstructed exposure of the tumor, with a piece of sterile rubber gloves employed to protect the brain surface (Figure 1(b)). Tumor debulking was then performed. After debulking the tumor, efforts were taken to dissect the tumor from adjacent structures such as cranial nerves, vessels, and brainstem and pursue a total resection of the tumor (Figures 1(c) and 1(d)).

**Figure 2:**
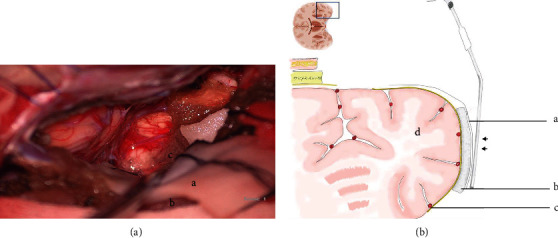
The surgicel-gelatin-cotton three-layer structure was used to protect the temporal lobe cortex from handheld suction retraction (black arrow) (C: surgicel as the first layer covered on the cortex; B: gelatin sponge as the middle layer that absorbs fluid and maintains the brain surface wet and flexible, providing a cushion against retraction; A: cotton on the top layer for fluid suction; D: the temporal lobe).

The surgicel-gelatin-cotton three-layer structure (surgicel as the first layer covered on the cortex, which perfectly fit yet do not stick to the cortex or injure the pia surface; gelatin sponge as the middle layer that absorbs fluid and maintains the brain surface wet and flexible, providing a cushion against retraction; cotton on the top layer for fluid suction) was used to protect the temporal lobe cortex from handheld suction retraction (Figure 2). Maintaining the brain's surface or veins wet and elastic by constant saline irrigation made them more tolerant to dynamic retraction of operating instruments. Maintain the arachnoidal plane and avoid crossover while dissecting the cranial nerves or veins. Retractorless surgery relied on the effective use of the handheld suction device and the operating instruments.

Electromyography (EMG) evoked potential monitoring system was used to monitor CN V and VII and the lower cranial nerves' function. Intraoperative brainstem auditory evoked potential (BAEP) monitoring was performed in all 29 cases.

## 3. Results

This study comprised 29 patients, 7 men and 22 women, with a median age of 56 years (range, 40-72 years). The median tumor diameter was 35 mm (range, 14-53 mm). In terms of clinical presentation, 19 (65.5%) cases developed CN dysfunction, and 10 cases developed other symptoms. The most common symptom was headache (*N* = 7, 24.1%), dizziness (*N* = 8, 27.6%), facial numbness (*N* = 6, 20.7%), gait disturbance (*N* = 5, 17.2%), and dysphagia (*N* = 5, 17.2%) (Table 1). Three (10.3%) patients developed obstructive hydrocephalus before the surgical treatment. There were 3 cases of recurrence PCMs with previous surgical treatment.

The PCMs in this study mainly involved the middle-upper clivus (*N* = 29, 100%), petroclinoid ligament (*N* = 26, 89.7%) petrous apex (*N* = 25, 86.2%), cerebellopontine angle (CPA) (*N* = 17, 58.6%), cavernous sinus (CS) (*N* = 10, 34.5%), middle fossa (*N* = 10, 34.5%), and internal acoustic canal (IAC) (*N* = 9, 31.0%) (Table 2).

All the procedures were completed without fixed retraction, mouth control microscope, or other specialized instrumentation such as a lighted suction. The median surgical time was 9.5 (6-15.5) hours. The gross total resection rate (Simpson grading I and II) was 82.8% (*N* = 24) (Table 1). Postoperative complications included cranial nerve palsy (*N* = 17, 58.6%), meningitis (*N* = 5, 17.2%), pulmonary infection (*N* = 4, 13.8%), hemiparesis (*N* = 2, 6.9%), brain stem infarction (*N* = 1, 3.4%), CSF leakage (3.4%), subscale hydrops (3.4%), hydrocephalus (3.4%), epidural hematoma (3.4%), and venous injury (3.4%). The incidence of temporal lobe injury was 3.4% (*N* = 1) and no epileptic seizures. Mean KPS 3 months postoperatively was 85.5 (Table 3).

**Figure 3:**
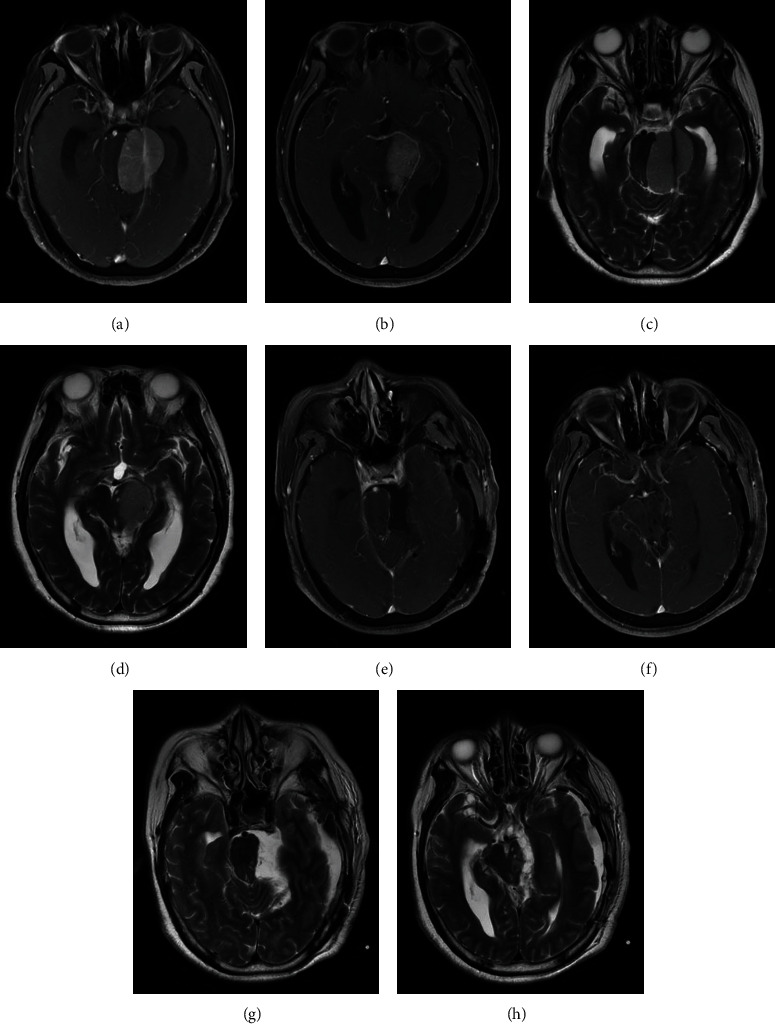
MRI obtained before and after surgical treatment with retractorless technics. (a, b) The preoperative enhanced MRI showed the tumor in the left petroclival region. The brainstem was squeezed to the right. (c, d) T2-weighted MRI showed no preoperative edema but two infarction lesions in the brain stem. (e, f) Postoperative enhanced MRI demonstrated a complete tumor resection. (g, h) Postoperative T2-weighted MRI showed no brain injury in the left temporal lobe.

Total resection of the tumor is pursued under the premise of preservation of neurological function (Figure 3).

## 4. Discussion

Retractorless surgery is widely accepted to reduce brain retraction injuries [1–4]. The first report of retractorless surgery by professor Spetzler in 2012 [5] inspired many neurosurgeons to carry out retractorless surgery, but also made some neurosurgeons flinch from a shortage of specially designed operative instruments as in the report. In this study, we retrospectively reported our results and technical details of retractorless surgery for PCM resection via subtemporal approach and found that all the procedures could be completed without fixed retraction or other specialized instruments. Patients with retractorless surgeries had a low incidence of temporal lobe injury (3.5%) and satisfactory tumor resection.

The petroclival area is a complex anatomical area because of its proximity to the brain stem, basilar artery, multiple dural folds, and traversing cranial nerves. The management of PCMs remains a formidable technical challenge for skull base surgeons [8–10]. Complete resection of these benign tumors is the only potentially curative option [10]. Reports on the gross total removal of PCMs ranged from 14.1% to 87.5% [9, 12, 13, 15–17, 20–23], mostly depending on the surgical approach and the size and extent of the tumor. The subtemporal approach is suitable for tumors located in the middle fossa or tumor invaded the cavernous sinus (CS), especially for regions from the upper two-thirds of the clivus to the petrous bone, even in cases with soft tumor tissue though part of the tumor located within the lower one-third clivus [15]. Postoperative temporal lobe injury is significantly associated with the subtemporal approach [14, 17, 18], and temporal lobe injury can lead to temporal lobe dysfunction such as seizure and aphasia, which can even worsen the patient's prognosis. However, the precise incident of temporal lobe injury for resection of PCMs is challenging to determine. The incidence of contusion or infarction associated with brain retraction reported to be probably 10% in skull base procedures [24] might be underestimated. Radiologically, ischemia beneath the retraction blade is reported to be noticed in up to 22% of patients in postoperative CT scans [25]. Retraction injuries were found in MRI scans in one-third (36%) of surgeries for ruptured anterior circulation aneurysms [26]. In our study, we found that with the application of retractorless technics for PCM resection, the incidence of temporal lobe injury (3.5%) was much lower than previous reports [24].

Retractorless surgery was firstly reported by professor Spetzler in 2012 [5]. A movement away from the use of fixed retractors has been heralded as an advance in skull base surgery, and an increasing number of retractorless surgeries are being performed. Professor Yashar [6] suggested that the utility of retractorless surgery should not be limited to skilled neurosurgeons, it could be performed safely even by young surgeons. On the other hand, as shown in the current reports of retractorless surgery [2, 5, 6], some cases still required the use of a fixed retractor, and the retractorless surgery was completed with the assistance of specialized operative instruments such as fiberoptic-lighted instruments and microscope with foot pedals or a mouthpiece, there are still some neurosurgeons standing by and watching, wondering if retractorless surgery can only be performed with chosen patients or if retractorless surgery can only be completed with specialized operative instruments. In this study, we found that all the procedures could be completed successfully without fixed retraction or other specialized instruments. Our experience (professor Xia Chengyu in our center has performed more than 400 consecutive cases of retractorless surgeries for treatment of complex skull base and deep-seated tumors including meningiomas, craniopharyngiomas, vestibular schwannomas, and hemangioblastomas, with routine operative instruments) shows that the concept of retractorless surgery could be used in all the patients with routine operative instruments. Whether using specialized instruments or not should not be the criterion for advanced surgical skills. However, we hope our study is an encouragement to neurosurgeons who want to carry out retractorless surgery yet flinch from a lack of specialized operative instruments, to embrace the retractorless concept as a way to improve surgery, and to minimize brain manipulation. The core concept of retractorless surgery should not merely be the abandonment of the fixed retraction but also a surgical technique requiring a series of appropriate comprehensive measures [2, 3, 5]. The retractorless surgery concept does not mean there is no retraction, but to replace a fixed retractor by using a handheld suction shaft and operating instrument as mobile retractors, which allows vascular structures to be mobilized to modify the operative corridor as needed and reduces retraction injury.

Brain relaxation (positioning, lumbar spinal drainage, and hyperventilation) and sufficient exposure via appropriate approach paved way for retractorless surgery. Adjacent brain cortical protection and appropriate surgical strategies facilitated retractorless surgery. To maintain the normal cortical elasticity and consistency is essential for the adjacent cortical protection. Kashimura et al. [19] developed a brain retraction technique by using gelatin sponge pieces to reduce retraction injury. The gelatin sponge retracts the temporal lobe along with the form of the cortical surface, acting more mildly than a fixed retractor. However, the gelatin sponge might stick to the cortex where the pia mater was injured. Shao [27] recommended a cottonoid slider, made by overlaying an adhesive plastic incision drape on one side of a dry cottonoid patty and trimming the edges to fit the form of the cottonoid, for gentle retraction to expose desired areas. Due to their smooth surface, the sliders can also glide across the parenchyma in an atraumatic manner to provide gentle retraction on the brain. Whereas the plastic incision drape stops fluid beneath to be transferred to the cotton covered on the drape by suction, here we recommend the surgicel-gelatin-cotton three-layer structure (Figure 2) for protection beneath the handheld suction. The surgicel as the first layer covered on the cortex, which perfectly fit yet do not stick to the cortex or injury the pia surface; gelatin sponge as the middle layer that absorbs fluid and maintains the brain surface wet and flexible, providing a cushion against retraction; cotton on the top layer for fluid suction.

As to surgical strategies, before full exposure of the tumor or tumor debulking, we prefer to cut off the tumor's blood supply by incising the tentorium along the tumor's posterior margin to the perimesencephalic cistern and along with the petrous erosion to the tumor's anterior margin, which requires minimal temporal lobe retraction. Then remove the bone of the petrous apex to gain an unobstructed exposure of the tumor through the above surgical corridor, which could partly free the tumor and cut off the tumor's blood supply. After that, debulk the tumor from the lateral toward the mesial portion and dissect the tumor from adjacent structures.

This study's limitations include a small sample size and a lack of randomized controlled group. What is more, it has inherent limits as a retrospective case study.

## 5. Conclusion

Retractorless surgery via the subtemporal approach, which can be completed successfully with routine operative instruments, is a reliable surgical treatment for PCMs. Comprehensive measures include brain relaxation, sufficient exposure, brain cortical protection, and appropriate surgical strategies that can significantly reduce brain retraction-related temporal lobe injury. More researches about retractor brain injury are needed to perfect the retractorless techniques.

## Figures and Tables

**Table 1:** Characteristics of participants.

Characteristic		*N*
Sex	Male	7
	Female	22
Age median (range) (years)		56 (40-72)
Symptoms	Headache	7
	Dizziness	8
	Visual deterioration	1
	Diplopia	3
	Facial numbness	6
	Trigeminal neuralgia	1
	Facial paralysis	2
	Hearing loss	2
	Dysphagia	5
	Gait disturbance	5
	Limb weakness	1
Preoperative hydrocephalus		3
Tumor recurrence		3
Duration of symptoms, median (range) (months)		13 (1-96)
Diameter of tumor, median (range) (mm)		35 (14-53)
Surgical time, median (range) (hours)		9.5 (6-15.5)
Simpson grading *N* (%)	I	1 (3.5)
	II	23 (79.3)
	III	2 (6.9)
	IV	3 (10.3)
	V	0
WHO classification	I	27
	II	2
Preoperative mean KPS		89.3
Mean KPS 3 months postoperatively		85.5

**Table 2:** Location and extension of PCMs.

Location and extension	
Middle-upper clivus	29
Lower clivus	0
Petrous apex	25
CS	10
CPA	17
Middle fossa	10
IAC	9
Petroclinoid ligament	26
BA encasement	2
Jugular foramen	0
Brainstem edema	4

CS: cavernous sinus; CPA: cerebellopontine angle; IAC: internal acoustic canal; BA: basilar artery.

**Table 3:** Postoperative complications.

Postoperative complications	*N* (%)
CN III palsy	5 (17.2)
CN IV palsy	5 (17.2)
CN V palsy	6 (20.7)
CN VI palsy	3 (10.3)
CN VII palsy	7 (24.1)
CN VII palsy	1 (3.4)
CN IX and X palsy	3 (10.3)
Hemiparesis	2 (6.9)
Meningitis	5 (17.2)
CSF leakage	1 (3.4)
Subscale hydrops	1 (3.4)
Hydrocephalus	1 (3.4)
Pulmonary infection	4 (13.8)
Brain stem infarction	1 (3.4)
Tracheotomy	3 (10.3)
Epidural hematoma	1 (3.4)
Epileptic seizures	0
Venous injury	1 (3.4)
Temporal lobe injury	1 (3.4)
Grade I	1 (3.4)
Grade II	0
Grade III	0
Grade IV	0

CN: cranial nerve; CSF: cerebrospinal fluid.

## Data Availability

The data that support the findings of this study are available from the corresponding author upon reasonable request.
